# Colistin Heteroresistance Is Largely Undetected among Carbapenem-Resistant *Enterobacterales* in the United States

**DOI:** 10.1128/mBio.02881-20

**Published:** 2021-01-26

**Authors:** Victor I. Band, Sarah W. Satola, Richard D. Smith, David A. Hufnagel, Chris Bower, Andrew B. Conley, Lavanya Rishishwar, Suzanne E. Dale, Dwight J. Hardy, Roberto L. Vargas, Ghinwa Dumyati, Marion A. Kainer, Erin C. Phipps, Rebecca Pierce, Lucy E. Wilson, Matthew Sorensen, Erik Nilsson, I. King Jordan, Eileen M. Burd, Monica M. Farley, Jesse T. Jacob, Robert K. Ernst, David S. Weiss

**Affiliations:** a Department of Microbiology and Immunology, Emory University, Atlanta, Georgia, USA; b Emory Vaccine Center, Atlanta, Georgia, USA; c Emory Antibiotic Resistance Center, Atlanta, Georgia, USA; d Division of Infectious Diseases, Department of Medicine, Emory University School of Medicine, Atlanta, Georgia, USA; e Georgia Emerging Infections Program, Atlanta, Georgia, USA; f Department of Microbial Pathogenesis, University of Maryland—Baltimore, Baltimore, Maryland, USA; g Center for Integrated Genomics, Georgia Institute of Technology, Atlanta, Georgia, USA; h ACM Global Laboratories, Rochester, New York, USA; i Department of Microbiology and Immunology, University of Rochester Medical Center, Rochester, New York, USA; j Department of Pathology and Laboratory Medicine, Rochester General Hospital, Rochester, New York, USA; k Infectious Diseases Division, University of Rochester Medical Center, Rochester, New York, USA; l Center for Community Health and Prevention, University of Rochester Medical Center, Rochester, New York, USA; m Tennessee Department of Health, Nashville, Tennessee, USA; n University of New Mexico, Albuquerque, New Mexico, USA; o New Mexico Emerging Infections Program, Albuquerque, New Mexico, USA; p Public Health Division, Oregon Health Authority, Salem, Oregon, USA; q Maryland Department of Health and Mental Hygiene, Baltimore, Maryland, USA; r Pataigin, LLC, Seattle, Washington, USA; s Department of Pathology and Laboratory Medicine, Emory University, Atlanta, Georgia, USA; t Research Service, Atlanta VA Medical Center, Decatur, Georgia, USA; Louis Stokes Veterans Affairs Medical Center

**Keywords:** colistin, heteroresistance, CRE, antibiotic resistance, polymyxins, *Enterobacterales*, *Enterobacteriaceae*

## Abstract

Heteroresistance is a form of antibiotic resistance where a bacterial strain is comprised of a minor resistant subpopulation and a majority susceptible subpopulation. We showed previously that colistin heteroresistance can mediate the failure of colistin therapy in an *in vivo* infection model, even for isolates designated susceptible by clinical diagnostics. We sought to characterize the extent of colistin heteroresistance among the highly drug-resistant carbapenem-resistant *Enterobacterales* (CRE). We screened 408 isolates for colistin heteroresistance. These isolates were collected between 2012 and 2015 in eight U.S. states as part of active surveillance for CRE. Colistin heteroresistance was detected in 10.1% (41/408) of isolates, and it was more common than conventional homogenous resistance (7.1%, 29/408). Most (93.2%, 38/41) of these heteroresistant isolates were classified as colistin susceptible by standard clinical diagnostic testing. The frequency of colistin heteroresistance was greatest in 2015, the last year of the study. This was especially true among *Enterobacter* isolates, of which specific species had the highest rates of heteroresistance. Among Klebsiella pneumoniae isolates, which were the majority of isolates tested, there was a closely related cluster of colistin-heteroresistant ST-258 isolates found mostly in Georgia. However, cladistic analysis revealed that, overall, there was significant diversity in the genetic backgrounds of heteroresistant K. pneumoniae isolates. These findings suggest that due to being largely undetected in the clinic, colistin heteroresistance among CRE is underappreciated in the United States.

## Abstract (precis)

Heteroresistance is an underappreciated phenomenon that may be the cause of some unexplained antibiotic treatment failures. Misclassification of heteroresistant isolates as susceptible may lead to inappropriate therapy.

## OBSERVATION

Increasing antibiotic resistance has been recognized as a major health threat by the Centers for Disease Control and Prevention (CDC) and World Health Organization, resulting in at least 35,000 deaths and 2.8 million infections annually in the United States (1). To combat infections due to highly resistant bacteria, such as carbapenem-resistant *Enterobacterales* (CRE), that have up to a 40% mortality rate (2), clinicians are increasingly turning to drugs of last resort, including the polymyxin antibiotic colistin (polymyxin E) (3). However, resistance even to last-line drugs is increasing (4–6). Further complicating efforts to combat multidrug-resistant bacteria are instances of treatment failure of strains classified as susceptible to a given antibiotic. Heteroresistance is a form of resistance in which a strain harbors both an antibiotic-resistant subpopulation and a majority population of susceptible cells. We recently demonstrated that colistin heteroresistance can lead to colistin treatment failure in an *in vivo* mouse model of infection with multiple *Enterobacter* and *Klebsiella* clinical isolates (7, 8). Furthermore, colistin heteroresistance may not be detected by traditional clinical testing methods. Failure to identify heteroresistance in the clinical laboratory may lead to treatment failures in serious CRE infections. We performed a retrospective study among multidrug-resistant CRE isolates collected between 2012 and 2015 to determine the frequency of colistin heteroresistance within this collection.

**FIG 1:**
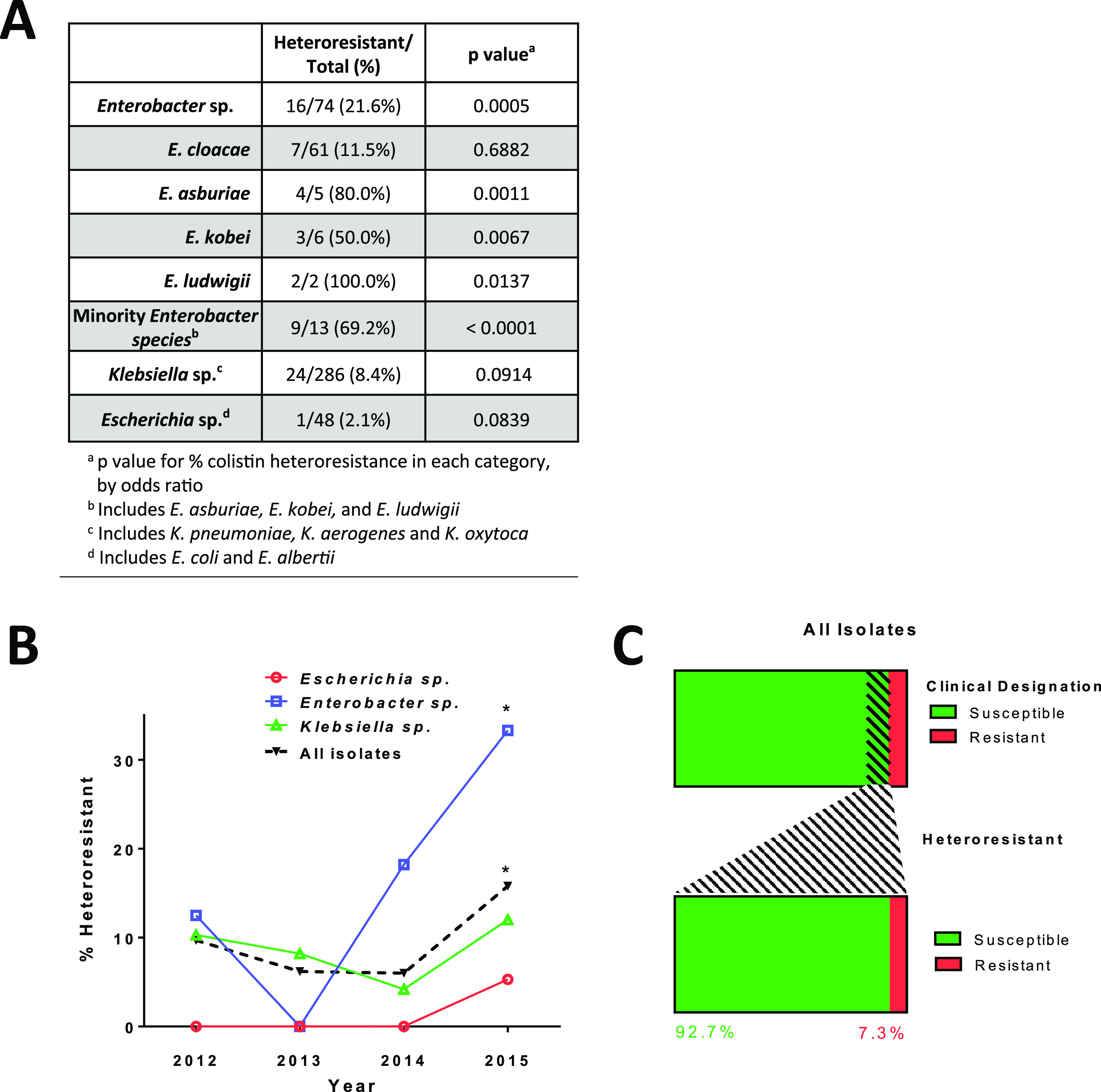
Rate of colistin heteroresistance by genus, year, and clinical detection. (A) Table of colistin heteroresistance incidence by the indicated genus, species, and *P* value for the odds ratio in each category. (B) Incidence of total colistin-heteroresistant isolates as a percentage in each year from 2012 to 2015. *, *P* < 0.05 for the positive linear trend from 2012 to 2015 (Cochran-Armitage trend test). (C) Proportion of isolates designated colistin susceptible (green) or colistin resistant (red) by standard clinical susceptibility testing. The portion with hatched lines indicates isolates that were heteroresistant by population analysis profile. The right margin represents the clinical designations of all colistin-heteroresistant isolates.

Four hundred eight CRE isolates included in this project were collected as part of the U.S. CDC Emerging Infections Program’s Multisite Gram-Negative Surveillance Initiative (MuGSI) (9, 10), an ongoing, active, population- and laboratory-based surveillance system for CRE isolated from urine and sterile sites, such as blood. Isolates were collected from clinical laboratories in metropolitan areas in eight U.S. states and adhered to a strict definition of CRE based on susceptibility testing and species identification (9). All isolates that fit this definition within the surveillance area during the study years of 2012 to 2015 were included in this study. All isolates are summarized in Table S1 in the supplemental material and belonged to 3 genera: *Klebsiella*, *Enterobacter*, and *Escherichia* (Fig. 1a). They originated from various culture sources (Table S1) and displayed resistance to last-line antibiotics, such as aminoglycosides and tigecycline (Table S2).

10.1128/mBio.02881-20.3 TABLE S1 Sex, age, and culture source of carbapenem-resistant *Enterobacterales.* Download Table S1, PDF file, 0.1 MB. Copyright © 2021 Band et al. 2021 Band et al. This content is distributed under the terms of the Creative Commons Attribution 4.0 International license.

10.1128/mBio.02881-20.4 TABLE S2 Susceptibility to last-line antibiotics by clinical testing. Download Table S2, PDF file, 0.10 MB. Copyright © 2021 Band et al. 2021 Band et al. This content is distributed under the terms of the Creative Commons Attribution 4.0 International license.

To detect heteroresistance to colistin, all isolates were tested via the population analysis profile (PAP) method. This consists of plating overnight cultures of each isolate onto solid Muller-Hinton (MH) agar with or without colistin concentrations of 0.5, 1, 2, 4, 16, 32, and 100 µg/ml. Surviving colonies were enumerated and used to detect colistin-resistant subpopulations characteristic of heteroresistance. Overall, PAP revealed a proportion of 10.1% (41/408 isolates) colistin heteroresistance (Fig. 1a), which was found among all genera tested. Compared to the proportion of isolates demonstrating heteroresistance, there was a lower proportion of isolates demonstrating “conventional” colistin resistance (7.1%, 29/408), in which all cells within the population exhibited a resistant phenotype. Additionally, the proportion of isolates with colistin heteroresistance was highest in the most recent year of the study (2015, 15.8%, 24/152, *P* = 0.0039, odds ratio = 2.636, 95% confidence interval [CI] = 1.366 to 5.087), and proportions were significantly higher than in all previous years (9.8% [4/41] in 2012, 6.2% [5/81] in 2013, 6.0% [8/134] in 2014) (Fig. 1b). Although only four sites collected isolates during all 4 years, including only those three sites, the rate of colistin heteroresistance in 2015 was still significantly higher than in prior years (17/118, 14.4%, *P* = 0.0135, odds ratio = 2.665, 95% CI = 1.225 to 5.799).

Of the 41 colistin-heteroresistant isolates detected by PAP, only 7.3% (3/41) were classified as colistin resistant by broth microdilution (BMD), while the vast majority (92.7%, 38/41) were classified as colistin susceptible (Fig. 1c). Overall, BMD classified 32 of the 408 CRE isolates as colistin nonsusceptible, a 7.2% rate of nonsusceptibility (including 3 heteroresistant isolates and 29 resistant isolates). However, testing by PAP revealed that the proportion of colistin-nonsusceptible isolates was actually 17.1% (70/408, 41 heteroresistant and 29 resistant isolates), which is more than double the rate detected by standard clinical testing (Table S3).

10.1128/mBio.02881-20.5 TABLE S3 Detection of colistin nonsusceptibility in colistin-heteroresistant *Enterobacterales* by standard clinical testing. Download Table S3, PDF file, 0.1 MB. Copyright © 2021 Band et al. 2021 Band et al. This content is distributed under the terms of the Creative Commons Attribution 4.0 International license.

*Enterobacter* spp. displayed the highest proportion of colistin heteroresistance (21.6%, 16/74, *P* = 0.0005, odds ratio = 3.410, 95% CI = 1.709 to 6.758), followed by *Klebsiella* spp. (8.4%, 24/286) and *Escherichia* (2.1%, 1/47). Among *Enterobacter*, the proportion of colistin heteroresistance was significantly higher in 2015 (33.3%, 11/33, *P* = 0.0338, odds ratio = 3.600, 95% CI = 1.103 to 11.748) than in prior years (12.2%, 5/41 from 2012 to 2014) (Fig. 1b). To further examine the rate of colistin heteroresistance among *Enterobacter* isolates in the collection, we stratified by species (as determined by the clinical laboratory). *Enterobacter* isolates included E. asburiae, E. cloacae, E. kobei, and E. ludwigii. Of these, E. cloacae (82.4%, 61/74) was the most common (Fig. 1a). The less-common species (designated here as “minority *Enterobacter* species”), *E. kobei* (8.1%, 6/74), *E. asburiae* (6.8%, 5/74), and *E. ludwigii* (2.7%, 2/74), accounted for the remaining *Enterobacter* isolates. The rate of colistin heteroresistance was highest among these minority *Enterobacter* species, with heteroresistance observed in 69.2% (9/13, *P* < 0.0001, odds ratio = 17.357, 95% CI = 4.209 to 71.576) of these isolates, compared to 11.5% (7/61) of E. cloacae isolates (Fig. 1a). Potentially in agreement with these findings, a previous report described variation in colistin heteroresistance rates among isolates from different Enterobacter cloacae complex genomic clusters (11). At least one colistin-heteroresistant *Enterobacter* isolate was identified from each of the eight study sites, indicating that *Enterobacter* isolates exhibiting this resistance phenotype were present in a wide distribution of geographic sites within the United States.

**FIG 2:**
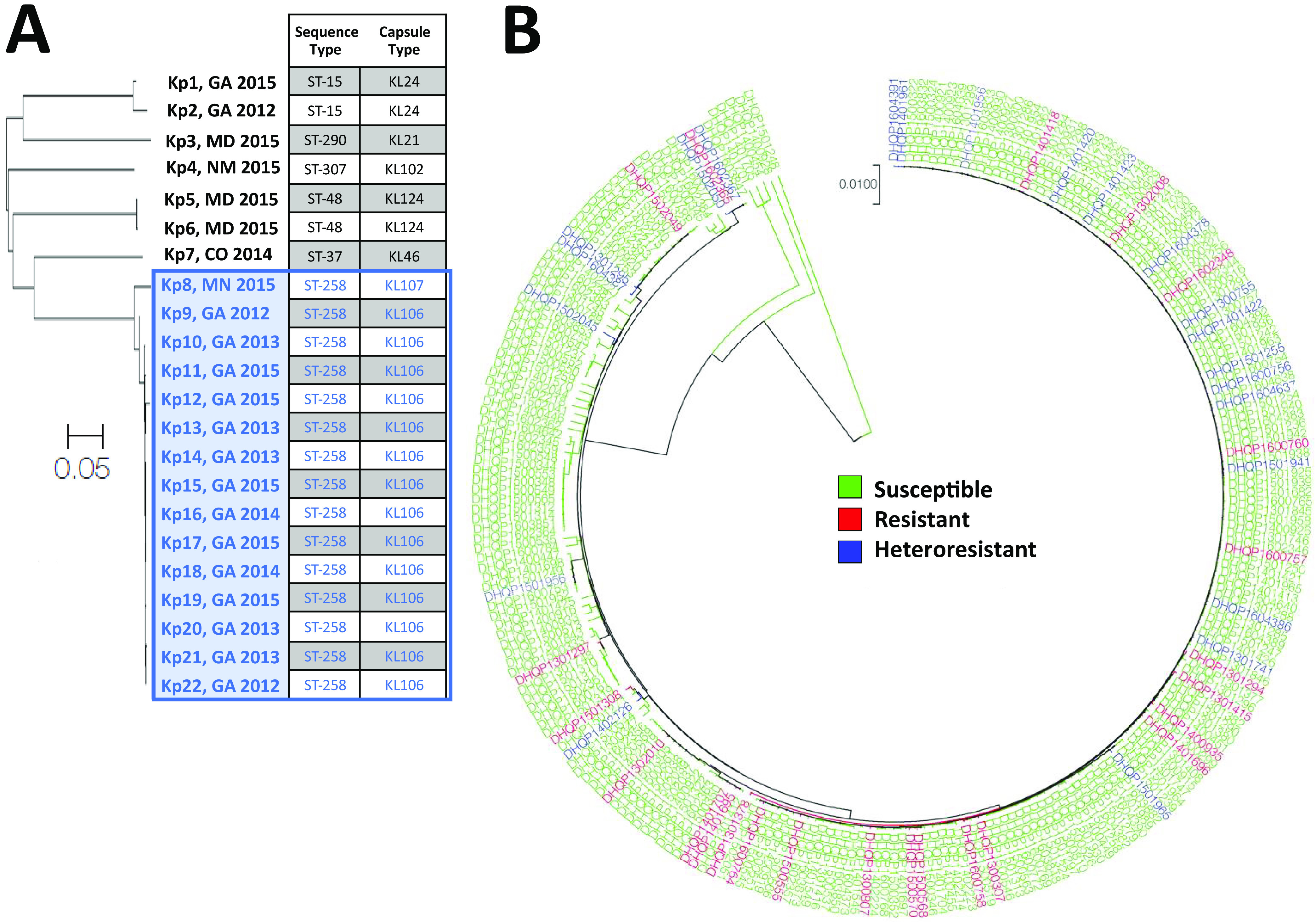
Colistin heteroresistance in K. pneumoniae occurs in genetically diverse isolates and forms a cluster of related isolates in Georgia. (A) Cladistic analysis of colistin-heteroresistant K. pneumoniae isolates by whole-genome sequence, with the corresponding sequence type and capsule type for each isolate. A closely related cluster of ST-258 isolates is highlighted in blue. (B) Cladistic analysis of all K. pneumoniae isolates in the study.

In contrast, *Klebsiella* isolates that were heteroresistant to colistin were found in only 5 states, with the majority (66.7%, 16/24) originating in the state of Georgia. Additionally, the proportion of heteroresistance in *Klebsiella* isolates was significantly higher in Georgia (15.0%, 16/107, *P* = 0.0034, odds ratio = 3.758, 95% CI = 1.550 to 9.115) than the proportion among isolates from all other states combined (4.5%, 8/179) (Table S4). The predominant *Klebsiella* species was K. pneumoniae, making up 90.9% of isolates, while other species included K. aerogenes and K. oxytoca. The higher rate of colistin heteroresistance among *Klebsiella* isolates in Georgia led us to consider whether this might be due to the presence of a predominant strain. To address this, we used cladistic analysis to determine the genetic relatedness of the colistin-heteroresistant K. pneumoniae isolates based on their whole-genome sequences (Fig. 2). This analysis revealed that there was a cluster of 15 closely related isolates within a 0.05 *P* distance (Fig. 2a, blue box) that were all sequence type 258 (ST-258) and contained similar antibiotic resistance genes. This genetic branch included isolates from all 4 years of the study, which did not cluster together temporally. The cluster of isolates consisted of 14 from Georgia and one from Minnesota, indicating that there was a highly related cluster of colistin-heteroresistant *Klebsiella* isolates present in Georgia. Among all the K. pneumoniae strains in the study, however, there was no association between whole-genome sequence and colistin susceptibility status (Fig. 2b). Taken together, the data indicate that colistin heteroresistance is a widespread phenomenon and that the presence of a cluster of heteroresistant K. pneumoniae isolates in Georgia may explain the high rate of colistin heteroresistance in this state.

10.1128/mBio.02881-20.6 TABLE S4 States of origin of carbapenem-resistant *Enterobacterales.* Download Table S4, PDF file, 0.1 MB. Copyright © 2021 Band et al. 2021 Band et al. This content is distributed under the terms of the Creative Commons Attribution 4.0 International license.

Previous studies have shown that colistin heteroresistance can be mediated by PhoPQ/PmrAB-dependent cationic sugar modifications to the lipid A component of lipopolysaccharide (LPS) (12–15), resulting in an increased charge of the Gram-negative bacterial outer membrane and reduced susceptibility to colistin, a cationic antimicrobial peptide. Lipid A was analyzed using a new extraction method termed fast lipid analysis technique (FLAT) (16), as well as the previously used El Hamidi et al. method (17), on a representative sample of 12 colistin-heteroresistant isolates from this study using matrix-assisted laser desorption ionization–time of flight (MALDI-TOF) mass spectrometry in the negative-ion mode (Table S5). After culture in the presence of colistin, which enriches for the resistant subpopulation, we observed an aminoarabinose (Δ*m/z* 131 mass shift) modification to the terminal phosphate in all heteroresistant isolates evaluated, as well as an additional phosphoethanolamine (Δ*m/z* 123 mass shift) modification in the Escherichia coli isolate analyzed (Fig. S1). These data further confirm that specific lipid A modifications are strongly associated with colistin heteroresistance, in agreement with previous studies (7, 18). Interestingly, MALDI-TOF mass spectrometry was able to detect lipid A modification in 9 out of 12 heteroresistant isolates even when the cultures were not enriched for resistant cells by growth in colistin (Table S5). These data indicate that MALDI-TOF approaches, which will be investigated further in future studies, may have diagnostic utility in detecting colistin heteroresistance which was missed by other diagnostic techniques.

10.1128/mBio.02881-20.2 FIG S1 Representative mass spectrometry analysis of lipid A. Sample EC 1 was cultured without (top) or with (bottom) 4 μg/ml colistin and then analyzed by MALDI-TOF mass spectrometry using the FLAT preparation method. Shifts in peaks indicated in the bottom panel represent additions of phosphoethanolamine (PetN, Δ123 *m/z*) and aminoarabinose (Ara4N, Δ131 *m/z*). Download FIG S1, PDF file, 0.1 MB. Copyright © 2021 Band et al. 2021 Band et al. This content is distributed under the terms of the Creative Commons Attribution 4.0 International license.

10.1128/mBio.02881-20.7 TABLE S5 Lipid A modifications of colistin HR isolates. Download Table S5, PDF file, 0.1 MB. Copyright © 2021 Band et al. 2021 Band et al. This content is distributed under the terms of the Creative Commons Attribution 4.0 International license.

This is the first multisite surveillance study for colistin heteroresistance among CRE in the United States. Treatment of highly antibiotic-resistant CRE relies on last-line drugs, including colistin, and the high rate of colistin heteroresistance may threaten the effective use of this antibiotic. The proportion of heteroresistance to colistin exceeded the proportion of “conventional” homogenous resistance, which, taken together, indicates that colistin nonsusceptibility is much more common than previously appreciated. Additionally, the vast majority of colistin-heteroresistant isolates were designated susceptible by standard clinical testing. The inability to detect colistin heteroresistance in the majority of heteroresistant isolates may lead to inappropriate treatment with colistin and might be a significant cause of unexplained antibiotic treatment failure. Detection of heteroresistance in these isolates was possible using the PAP method, which is both labor- and time-intensive, making it an infeasible diagnostic method to employ in a clinical setting. Improvements in susceptibility testing are pivotal to improving clinical detection of heteroresistance, as observed using MALDI-TOF mass spectrometry in this study. Overall, this study shows that colistin heteroresistance is an underrecognized phenomenon among CRE in the United States.

10.1128/mBio.02881-20.1 TEXT S1 Detailed materials and methods of techniques used in this work. Download Text S1, DOCX file, 0.02 MB. Copyright © 2021 Band et al. 2021 Band et al. This content is distributed under the terms of the Creative Commons Attribution 4.0 International license.
