# Supplementary material for: Colistin Heteroresistance Is Largely Undetected among Carbapenem-Resistant Enterobacterales in the United States
Source: mBio. 2021 Jan 26;12(1):e02881-20. doi: 10.1128/mBio.02881-20 (PMC7858057; doi:10.1128/mBio.02881-20)
Supplement: TABLE S1 [file mBio.02881-20-st001.pdf]

Supplemental Table 1. Sex, Age, and Culture Source of Carbapenem Resistant *Enterobacterales*

	Colistin susceptibility by PAP, No. (%)				
	Susceptible	Conventional Resistant	Heteroresistant	Total	p value[a]
**Sex**					
**Female**	194 (86)	16 (7)	16 (7)	226	
**Male**	143 (79)	13 (7)	24 (13)	180	0.0439
**Age**					
**0-9**	1 (25)	0 (0)	3 (75)	4	0.0033
**10-19**	2 (100)	0 (0)	0 (0)	2	1.0000
**20-29**	14 (88)	2 (13)	0	16	0.3856
**30-39**	17 (85)	1 (5)	2 (10)	20	1.0000
**40-49**	27 (93)	1 (3)	1 (3)	29	0.3391
**50-59**	65 (87)	3 (4)	7 (9)	75	1.0000
**60-69**	92 (82)	8 (7)	12 (11)	112	0.7120
**70-79**	72 (87)	9 (11)	2 (2)	83	0.0071
**80+**	47 (72)	5 (8)	13 (20)	65	0.0056
**Culture source**					
**Urine**	275 (83)	24 (7)	34 (10)	333	1.0000
**Blood**	47 (82)	5 (9)	5 (9)	57	1.0000
**Other sterile site**	13 (87)	0 (0)	2 (13)	15	0.6561
**Total**	338	29	41	408	

[a] p value for % colistin heteroresistance in each category, by odds ratio

PAP – population analysis profile
