# Supplementary material for: Colistin Heteroresistance Is Largely Undetected among Carbapenem-Resistant Enterobacterales in the United States
Source: mBio. 2021 Jan 26;12(1):e02881-20. doi: 10.1128/mBio.02881-20 (PMC7858057; doi:10.1128/mBio.02881-20)
Supplement: TABLE S2 [file mBio.02881-20-st002.pdf]

Supplemental Table 2. Susceptibility to Last-line Antibiotics by Clinical Testing

	Colistin Susceptibility by PAP, No. Non-susceptible/Total Tested (%)				
	Susceptible	Conventional Resistant	Heteroresistant	Total	p value[a]
**Amikacin Non-susceptible**	107/300 (35.6)	14/25 (56.0)	10/36 (27.8)	131/361 (36.3)	0.2659
**Gentamicin Non-susceptible**	120/311 (38.6)	15/25 (60.0)	8/38 (21.1)	143/374 (38.2)	0.0254
**Tobramycin Non-susceptible**	223/302 (73.8)	19/24 (79.2)	22/35 (62.9)	264/361 (73.1)	0.1527
**Tigecycline Non-susceptible**	4/62 (6.5)	1/5 (20.0)	1/13 (7.7)	6/80 (7.5)	0.9771

[a] p value for % colistin heteroresistance in each category, by odds ratio

PAP – population analysis profile
