# Supplementary material for: Colistin Heteroresistance Is Largely Undetected among Carbapenem-Resistant Enterobacterales in the United States
Source: mBio. 2021 Jan 26;12(1):e02881-20. doi: 10.1128/mBio.02881-20 (PMC7858057; doi:10.1128/mBio.02881-20)
Supplement: TABLE S3 [file mBio.02881-20-st003.pdf]

Supplemental Table 3. Detection of Colistin Non-susceptibility in Colistin Heteroresistant *Enterobacterales* by Standard Clinical Testing

	Total Isolates Detected by BMD, No. (%)			Total Isolates, No. (%)		
	Detected Conventional Resistance[a]	Detected Heteroresistance[b]	Total Lab Detected Non-susceptible[c]	Undetected Heteroresistance[d]	Total Non-Susceptible[e]	Change in Non-susceptible Rate[f]
***Enterobacter* species**	2 (2.7)	1 (1.4)	3 (4.1)	15 (20.3)	18 (24.3)	+600%
***Escherichia* species**	0 (0.0)	0 (0.0)	0 (0.0)	1 (2.1)	1 (2.1)	n/a
***Klebsiella* species**	27 (9.4)	2 (0.7)	29 (10.1)	22 (7.7)	51 (17.8)	+176%
**Total**	29 (7.1)	3 (0.7)	32 (7.8)	38 (9.3)	70 (17.1)	+219%

[a] Total conventional resistant isolates detected as non-susceptible by broth-microdilution
[b] Total heteroresistant isolates detected as non-susceptible by broth microdilution
[c] Total isolates (resistant and heteroresistant) detected as non-susceptible by broth microdilition
[d] Total heteroresistant isolates detected as susceptible by broth microdilution
[e] Total non-susceptible isolates as detected by laboratory based population analysis profile
[f] Change in rate of non-susceptibility, in %, as detected by population analysis profile compared to broth-microdilution
