# Supplementary material for: Colistin Heteroresistance Is Largely Undetected among Carbapenem-Resistant Enterobacterales in the United States
Source: mBio. 2021 Jan 26;12(1):e02881-20. doi: 10.1128/mBio.02881-20 (PMC7858057; doi:10.1128/mBio.02881-20)
Supplement: TABLE S4 [file mBio.02881-20-st004.pdf]

Supplemental Table 4. State of Origin of Carbapenem Resistant *Enterobacterales*

	Colistin susceptibility by PAP, No. (%)				
	Susceptible	Conventional Resistant	Heteroresistant	Total	p value[a]
**State**					
**Colorado**	35 (88)	2 (5)	3 (8)	40	0.7833
**Georgia**	101 (80)	8 (6)	17 (13)	126	0.1530
**Maryland**	104 (87)	11 (9)	5 (4)	120	0.0107
**Minnesota**	49 (88)	3 (5)	4 (9)	56	0.6316
**New Mexico**	8 (80)	0 (0)	2 (20)	10	0.2653
**New York**	23 (82)	1 (4)	4 (14)	28	0.5085
**Oregon**	11 (61)	4 (22)	3 (17)	18	0.4091
**Tennessee**	7 (70)	0 (0)	3 (30)	10	0.0687

[a] p value for % colistin heteroresistance in each category, by odds ratio
PAP – population analysis profile
