# Supplementary material for: Colistin Heteroresistance Is Largely Undetected among Carbapenem-Resistant Enterobacterales in the United States
Source: mBio. 2021 Jan 26;12(1):e02881-20. doi: 10.1128/mBio.02881-20 (PMC7858057; doi:10.1128/mBio.02881-20)
Supplement: FIG S1 [file mBio.02881-20-sf001.pdf]

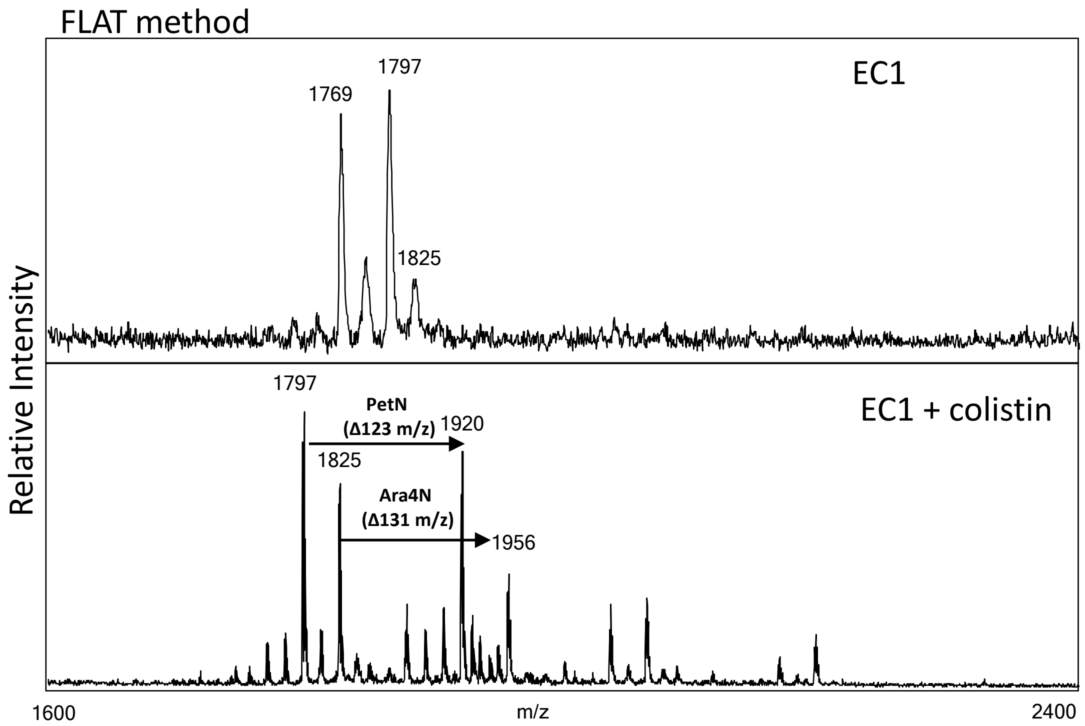

Supplemental Figure 1. Representative mass spectrometry analysis of lipid A. Sample EC 1 cultured without (top) or with (bottom) 4ug/mL colistin, and then analyzed by MALDI-TOF using the FLAT preparation method. Shifts in peaks indicated on the bottom panel represent additions of phosphoethanolamine (PetN, Δ123m/z) and aminoarabinose (Ara4N, Δ131m/z)
