# Supplementary material for: Colistin Heteroresistance Is Largely Undetected among Carbapenem-Resistant Enterobacterales in the United States
Source: mBio. 2021 Jan 26;12(1):e02881-20. doi: 10.1128/mBio.02881-20 (PMC7858057; doi:10.1128/mBio.02881-20)
Supplement: TABLE S5 [file mBio.02881-20-st005.pdf]

Supplemental Table 5. Lipid A modifications of colistin HR isolates.

Strain	Species	FLAT method		Caroff method	
		- Colistin	+ Colistin	- Colistin	+ Colistin
K1	*Klebsiella pneumoniae*	Ara4N	Ara4N	Ara4N	Ara4N
K2	*Klebsiella pneumoniae*	Ara4N	Ara4N	Ara4N	Ara4N
K3	*Klebsiella pneumoniae*				Ara4N
K4	*Klebsiella pneumoniae*		Ara4N		Ara4N
K5	*Klebsiella aerogenes*		Ara4N		Ara4N
K6	*Klebsiella pneumoniae*	Ara4N	Ara4N	Ara4N	Ara4N
K7(R)	*Klebsiella pneumoniae*	Ara4N	Ara4N	Ara4N	Ara4N
E1	*Enterobacter asburiae*	Ara4N	Ara4N	Ara4N	Ara4N
E2	*Enterobacter ludwigii*		Ara4N	Ara4N	Ara4N
E3	*Enterobacter kobei*	Ara4N	Ara4N		Ara4N
E4	*Enterobacter ludwigii*		Ara4N	Ara4N	Ara4N
E5	*Enterobacter cloacae*		Ara4N	Ara4N	Ara4N
EC1	*Escherichia coli*		Ara4N, PetN		Ara4N, PetN

Isolates were grown in both antibiotic free media (- colistin) and media containing 4ug/mL colistin (+ colistin) to select for the resistant subpopulation.
Isolate K7(R) was homogenously resistant to colistin, all other isolates are colistin heteroresistant.
Ara4N – aminoarabinose
PetN – phosphoethanolamine
