# Supplementary material for: Colistin Heteroresistance Is Largely Undetected among Carbapenem-Resistant Enterobacterales in the United States
Source: mBio. 2021 Jan 26;12(1):e02881-20. doi: 10.1128/mBio.02881-20 (PMC7858057; doi:10.1128/mBio.02881-20)
Supplement: TEXT S1 [file mBio.02881-20-s0001.docx]

**Supplemental Methods**

Study Design

CRE isolates included in this project were collected as part of the U.S. Centers for Disease Control and Prevention (CDC) Emerging Infections Program’s Multi-site Gram-Negative Surveillance Initiative (MuGSI). MuGSI is an ongoing, active, population- and laboratory-based surveillance system for CRE. Isolates were collected between 2012 and 2015 from clinical laboratories in metropolitan areas in eight US sites: Colorado (2013-15), Georgia (2012-15), Maryland (2013-15), Minnesota (2012-15), New Mexico (2014-15), New York (2013-15), Oregon (2012-15) and Tennessee (2014-15). Susceptibility results from standard phenotypic antibiotic susceptibility testing methods (e.g., minimum inhibitory concentration (MIC) or, zone diameter interpretive criteria) used by the clinical laboratories determined eligibility for the project. *Enterobacter*, *Escherichia,* and *Klebsiella* species found to be non-susceptible to at least one carbapenem excluding ertapenem (doripenem, imipenem, meropenem with a MIC ≥2 µg/mL), and resistant to all third generation cephalosporins tested (ceftriaxone MIC ≥4 µg/mL, ceftazidime MIC ≥16 µg/mL, and cefotaxime MIC ≥4 µg/mL) were included. Clinical and demographic data collection was performed by surveillance epidemiologists at each MuGSI site. A convenience sample of isolates from incident CRE cases was collected from surveillance laboratories and sent to CDC for further characterization.

Colistin heteroresistance testing

Colistin heteroresistance was assessed using the population analysis profile (PAP) method [18]. Briefly, each isolate was grown from a single colony overnight in Mueller-Hinton (MH) broth and serial dilutions were plated on solid MH agar without colistin or containing colistin concentrations of 0.5, 1, 2, 4, 16, 32, and 100 ug/mL. The proportion of resistant colonies was calculated by dividing the number of colonies growing on each concentration of colistin by the number of total colonies growing on a plate with no colistin. Isolates were considered heteroresistant if the proportion (percentage) of resistant colonies at 16ug/mL of colistin or greater was at least 1 in 10^6^ colonies but less than 5 in 10^1^ colonies. Isolates were classified as exhibiting “conventional” resistance (wherein all cells of the population are phenotypically resistant) if the proportion of total colonies surviving at 4ug/mL colistin or greater was more than 5 in 10^1^ colonies. All other isolates were considered colistin susceptible. All tests were repeated twice to ensure consistent results.

Broth Microdilution

Colistin susceptibility testing using broth microdilution (BMD) was performed according to CLSI standards. 5x10^5^ colony forming units (CFU) were grown in Cation-Adjusted Mueller-Hinton in the presence of potency adjusted colistin methyl sulfate at 37˚C shaking at 250 rpm for 20 hours. For each isolate, the MIC was determined as the lowest concentration inhibiting visible growth. Isolates with an MIC of ≥4 were classified as resistant (non-wild type according to CLSI standards), while isolates with an MIC of ≤2 were classified as susceptible.

Whole Genomic Sequencing

Genome sequence data for the *Klebsiella* isolates analyzed here were accessed as FASTQ files from the National Center for Biotechnology Information (NCBI) Short Read Archive (SRA) (https://www.ncbi.nlm.nih.gov/sra). SRA accession numbers and isolate names are shown in Supplementary Table S2. The program FASTQC version 0.11.5 ^28^ was used to evaluate the quality of the FASTQ files. Low quality reads and bases (Q<20) were removed prior to assembly using the program PRINSEQ version 0.20.4 [19]. *Ab initio* genome assembly was performed using the program SPAdes version 3.10.0 [20] with default parameters. The resulting assemblies were screened for the presence of plasmids by using BLAT version 36x1 [21] sequence similarity searches against a custom database of bacterial plasmid sequences curated from the NCBI Genbank RefSeq database [22]. Plasmid size was confirmed by pulsed field gel electrophoresis (PFGE). Antibiotic resistance genes were annotated using BLAST [23] sequence comparisons with the ARG-ANNOT [24] and BIGSdb [25] databases, respectively. Pairwise genome sequence comparisons were performed using the MUMmer version 3.23 [26] implementation of the average nucleotide identity (ANI) approach. The resulting pairwise genome sequence identities were converted to p-distances, which were used to reconstruct the isolate phylogeny with the program MEGA version 6.0 [27].

Lipid A Analysis

Lipid A modifications were detected using the “FLAT” MALDI-TOF based method with two independent sample preparations. Briefly, microbial colony smears or liquid samples were applied to a specified target location on a stainless steel MALDI plate. 1µL of 70% citric acid extraction buffer, pH 4.5 was spotted on top of the plated bacteria and the target plate was incubated in a humidified chamber for 30 minutes at 110ºC. The MALDI plate was washed with deionized water from a squeeze bottle, allowed to air dry, then 1 µL of norharmane matrix solution was applied (10 mg/mL in 12:6:1, v/v/v chloroform/methanol/water) to each target location. Following the method of Leung *et al.* [28], spectra were acquired from target locations in negative ion mode using a Microflex LRF MALDI-TOF MS (Bruker, Billerica MA) in reflectron mode with a limited mass range of 1000-2400 *m/z.* Typically, 300 laser shots were summed to acquire each mass spectrum. Data were processed with flexAnalysis software (version 3.4). Similar lipid A results were observed using the lipid microextraction method (“Caroff” in Table S5) originally described by El Hamidi *et al* [16].

Statistical Analysis

All statistical analyses were conducted using SAS software (SAS University Edition, www.sas.com/en_us/software/university-edition.html). Proportion of heteroresistance was calculated by dividing the number of heteroresistant isolates by the number of total isolates, represented as a percentage. All p values were calculated using odds ratio (OR) calculation in SAS.

**Supplemental References**

18. Satola SW, Farley MM, Anderson KF, Patel JB. Comparison of detection methods for heteroresistant vancomycin-intermediate Staphylococcus aureus, with the population analysis profile method as the reference method. J Clin Microbiol **2011**; 49:177-83.

19. Schmieder R, Edwards R. Quality control and preprocessing of metagenomic datasets. Bioinformatics **2011**; 27:863-4.

20. Bankevich A, Nurk S, Antipov D, et al. SPAdes: a new genome assembly algorithm and its applications to single-cell sequencing. J Comput Biol **2012**; 19:455-77.

21. Kent WJ. BLAT--the BLAST-like alignment tool. Genome Res **2002**; 12:656-64.

22. Pruitt KD, Tatusova T, Brown GR, Maglott DR. NCBI Reference Sequences (RefSeq): current status, new features and genome annotation policy. Nucleic Acids Res **2012**; 40:D130-5.

23. Camacho C, Coulouris G, Avagyan V, et al. BLAST+: architecture and applications. BMC Bioinformatics **2009**; 10:421.

24. Gupta SK, Padmanabhan BR, Diene SM, et al. ARG-ANNOT, a new bioinformatic tool to discover antibiotic resistance genes in bacterial genomes. Antimicrob Agents Chemother **2014**; 58:212-20.

25. Jolley KA, Maiden MC. BIGSdb: Scalable analysis of bacterial genome variation at the population level. BMC Bioinformatics **2010**; 11:595.

26. Kurtz S, Phillippy A, Delcher AL, et al. Versatile and open software for comparing large genomes. Genome Biol **2004**; 5:R12.

27. Tamura K, Stecher G, Peterson D, Filipski A, Kumar S. MEGA6: Molecular Evolutionary Genetics Analysis version 6.0. Mol Biol Evol **2013**; 30:2725-9.

28. Leung LM, Fondrie WE, Doi Y, et al. Identification of the ESKAPE pathogens by mass spectrometric analysis of microbial membrane glycolipids. Sci. Rep. **2017**; 7:6403.
